# An Uncommon Occurrence of Rectal Leiomyoma: A Case Report and Literature Review

**DOI:** 10.7759/cureus.69644

**Published:** 2024-09-18

**Authors:** Claudia Nieuwland, Rafael Pajaro

**Affiliations:** 1 Internal Medicine, Morristown Medical Center, Morristown, USA; 2 Medical School, St. George's University School of Medicine, St. George's, GRD

**Keywords:** colonoscopy, colonoscopy surveillance, diarrhea, gastrointestinal leiomyoma, polyp, rectal leiomyoma, rectal tumor, smooth muscle tumor, uterine leiomyoma

## Abstract

Gastrointestinal (GI) leiomyomas, particularly those located in the rectum, are extremely rare, accounting for approximately 0.1% of rectal tumors. We report the case of a middle-aged female who presented with chronic diarrhea of unknown origin. A colonoscopy was performed, during which a rectal polyp was resected. Histopathology examination of the polyp revealed smooth muscle proliferation and a positive immunohistochemical profile for desmin, consistent with leiomyomatous nature and a diagnosis of rectal leiomyoma. This case underscores the importance of a comprehensive diagnostic approach, including endoscopic, histologic, and immunohistochemical analysis, to differentiate leiomyomas from other GI tumors. Following GI evaluation, the patient underwent a total abdominal hysterectomy, which revealed multiple uterine leiomyomas. The concurrent presence of rectal and uterine leiomyomas in this patient is significant and suggests a potential association between these lesions in females. Further research is needed to explore the potential relationship between rectal and uterine leiomyomas and its implications for clinical practice.

## Introduction

Routine colonoscopy screening is an important component in the prevention and early detection of colorectal cancer, a malignancy that ranks among the most prevalent worldwide [1]. This screening method can identify and remove pre-cancerous lesions, averting the progression into malignant lesions. Common pathologies encountered during colonoscopy screenings encompass various types of polyps, including adenomatous polyps, hyperplastic polyps, and serrated polyps [1]. Understanding the prevalence and characteristics of these lesions is fundamental in guiding management and further screening.

Leiomyomas, commonly referred to as fibroids, are benign smooth muscle tumors predominantly found within the uterine myometrium. Leiomyomas can also occur in the gastrointestinal (GI) tract, where they are termed GI leiomyomas. Similar to lesions in the uterus, GI leiomyomas are benign smooth muscle tumors originating from the muscularis mucosa [2]. They are classified as intestinal mesenchymal tumors alongside GI stromal tumors (GISTs) and leiomyosarcomas. Differentiating between GI leiomyomas, GISTs, and leiomyosarcomas is crucial, as their treatment and prognosis vary significantly; accurate histopathological confirmation is essential for appropriate diagnosis and management.

GI leiomyomas are rare compared to other GI tumors, accounting for approximately 1% of GI tract tumors [3]. They can occur throughout the GI tract but are most commonly found in the esophagus, stomach, and small bowel, with only about 3% located in the colon and rectum [2-4]. Among all types of specifically rectal tumors, leiomyomas are extremely rare, representing less than 0.1% of rectal tumors [3]. The finding of colon and rectal leiomyoma is more common in men, most often discovered in the fifth and sixth decades of life [5]. Of note, this is also the time in which most patients begin colonoscopy screening.

We report the case of a 5 mm rectal polyp that was identified and resected on colonoscopy and later found to be a leiomyoma on histological examination. The rare occurrence of a rectal leiomyoma, especially in a female, makes this case a valuable addition to the literature.

## Case presentation

A 46-year-old Hispanic female was referred to gastroenterology by her primary care provider for further evaluation of bloating and diarrhea. The diarrhea started approximately three months prior and had been worsening in the past month. At the time of the presentation, she had been having diarrhea after every meal. She had been unable to associate her symptoms with any particular food or other cause. She denied other symptoms such as nausea, vomiting, abdominal pain, hematochezia, or melena. She had no changes in weight and denied any nocturnal bowel movements. She had no recent illnesses, travel, or dietary changes. She had never had an upper endoscopy or colonoscopy before. Past medical history is significant for asthma, hypertension, gastroesophageal reflux disease (GERD), and *BRCA* positivity. At the time, the patient was scheduled for total abdominal hysterectomy and bilateral salpingo-oophorectomy due to abnormal uterine bleeding, as well as prophylaxis for a positive *BRCA 1*/*MSH6* variant. The physical examination was unremarkable. GI panel was negative for infections. *Helicobacter pylori* (*H. pylori*) stool antigen was negative. The fecal occult blood test was negative. Fecal calprotectin was found elevated to 64 μg/mg (Table 1), suggestive of possible intestinal inflammation.

**Table 1:** Pertinent lab investigations

Parameter	Result	Reference Range
*Helicobacter pylori* (*H. pylori*) antigen, stool	Negative	Negative
Fecal calprotectin	64	<50 μg/mg

The patient underwent both upper endoscopy and colonoscopy for further evaluation. Upper endoscopy was significant for diffuse mildly erythematous mucosa in the gastric antrum. A single 4 mm sessile polyp was also found in the gastric antrum. Biopsies were taken with cold forceps for histology. Pathology of the gastric polyp revealed a hyperplastic/regenerative polyp, and gastric antrum biopsies showed evidence of mild chronic gastritis. The immunohistochemical stain for H. pylori was negative.

**Figure 1:**
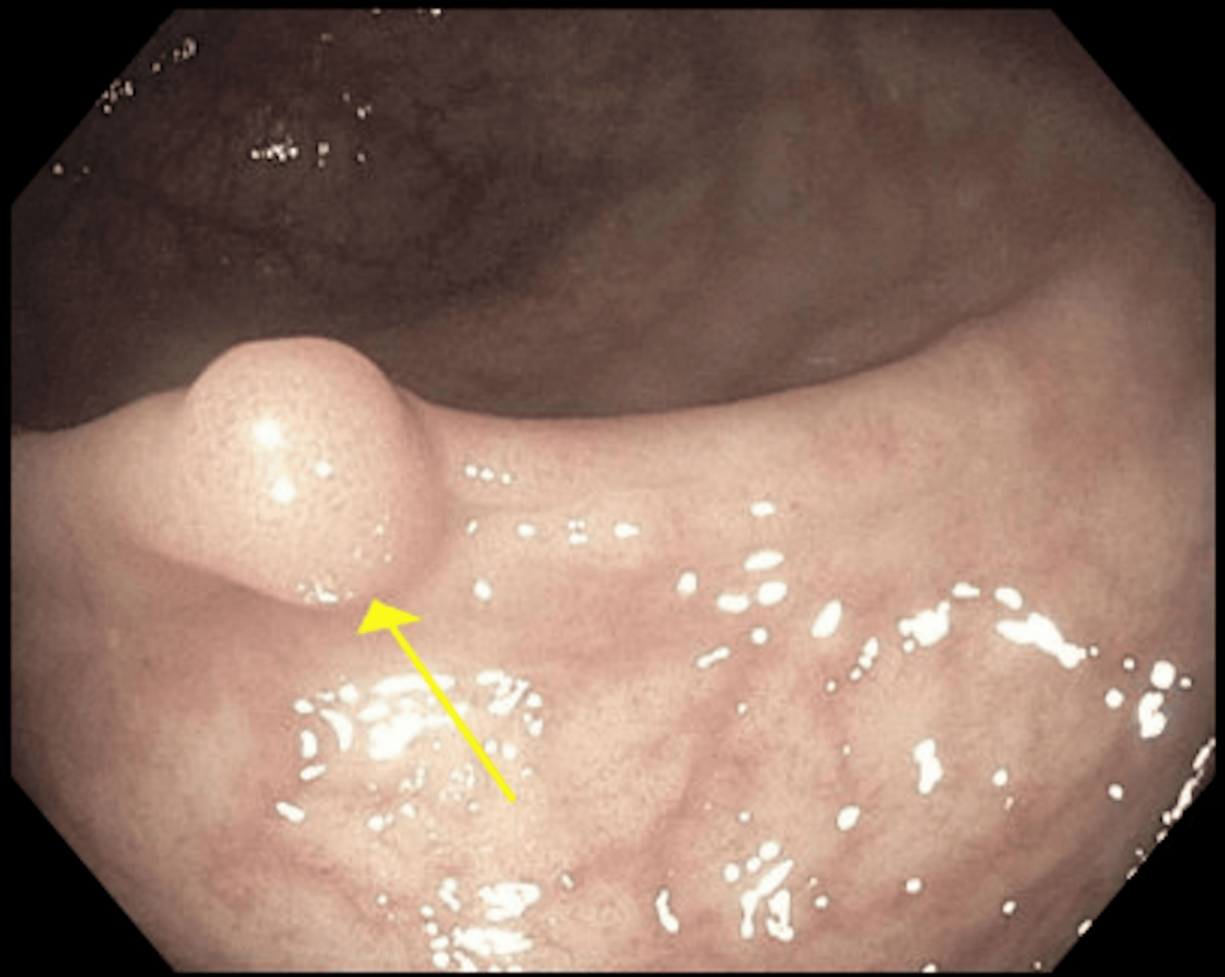
Colonoscopy showing a 5 mm sessile polyp in the rectum (arrow)

**Figure 2:**
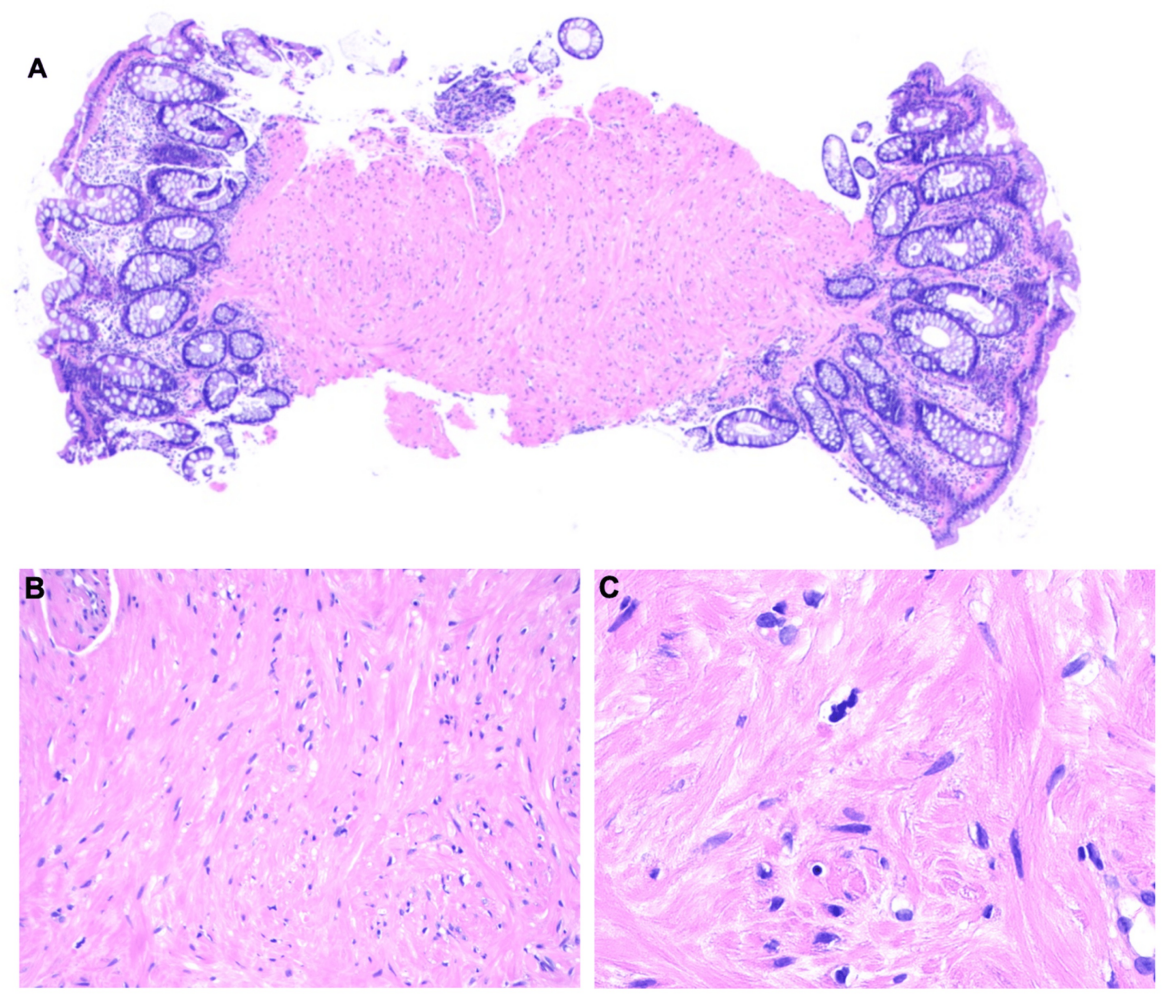
Histology of the rectal polyp (hematoxylin and eosin stain) showing smooth muscle proliferation consistent with leiomyoma (A) Low-power view (×20); (B) medium-power view (×200); (C) high-power view (×400)

**Figure 3:**
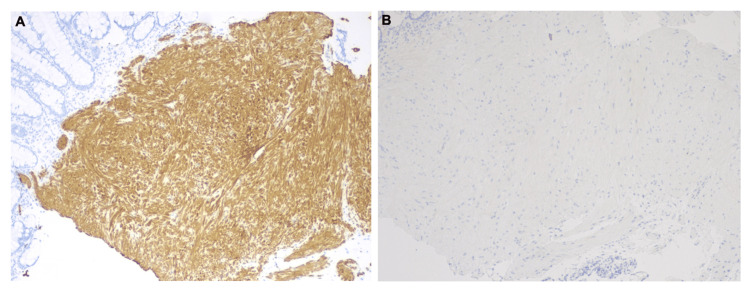
Immunohistochemical staining of the rectal polyp (A) Desmin positive; (B) DOG-1 negative

On colonoscopy, perianal and digital rectal examinations were normal. One 5 mm polyp, classified as sessile, was identified in the rectum (Figure 1), which was resected with a cold snare and removed. The examination was otherwise normal on direct and retroflexion views. Multiple biopsies were taken throughout the colon to rule out microscopic colitis. The examined portion of the ileum was normal. Pathology of the colon random biopsies showed no significant histopathologic changes. Pathology of the rectal polyp, however, revealed fragments of rectal mucosa with smooth muscle proliferation (Figure 2). The tissue exhibited positive immunohistochemical staining for the smooth muscle marker desmin, while GIST markers CD117, DOG-1, and S100 were negative, suggestive of leiomyoma (Figure 3).

The patient's symptoms resolved by the time of her follow-up appointment with her primary care provider a few weeks later. Four months later, the patient underwent a hysterectomy and bilateral salpingo-oophorectomy as planned for *BRCA* positivity, which revealed several leiomyomas of the uterus.

## Discussion

Leiomyomas of the colon and rectum typically manifest as solitary, well-defined polyps that are asymptomatic and, therefore, primarily discovered incidentally on routine colonoscopy. However, depending on the size and location of the lesion, patients may present symptomatically with abdominal pain, GI bleeding, bowel obstruction, or, rarely, intussusception [4,5]. In this case, the patient underwent endoscopic evaluation for seemingly unexplained GI symptoms, which seemed to resolve shortly after intervention. However, whether the endoscopic findings were causative of the presenting symptoms remains unknown.

Similar to the treatment of most polyps discovered on routine colonoscopy, complete resection with polypectomy is considered the standard, most definitive treatment for GI leiomyomas [4-6]. The diagnosis is made on histologic examination, as endoscopically, lesions are often indistinguishable from other types of tumors [2,4]. It is important that leiomyomas of the rectum be distinguished histologically from other types of polyps, GISTs, and leiomyosarcomas. Rarely do benign leiomyomas progress to leiomyosarcomas; however, the differentiation between these two lesion types, again, may be difficult and must be confirmed by pathological examination as the prognosis between tumor types differs greatly [3,7]. A complete resection of the lesion is often required to obtain an accurate histologic analysis and diagnosis [7]. This concept was highlighted in a case report of rectal leiomyosarcoma where the histological type of the tumor was not determined even after two biopsies of the rectum, but the final diagnosis was made on pathological analysis of the entire tumor, which revealed leiomyosarcoma [7]. The patient in the present case had a full resection of the lesion, which could be examined histologically, with no findings of concern for malignancy.

Currently, no official guidelines exist for the follow-up of GI leiomyomas. The general standard established in relevant literature is to have patients undergo repeat colonoscopies for continued surveillance [4,8]. The recommended time interval, however, is what remains of debate and, therefore, seems to be left to provider discretion. Although this continued surveillance is commonly performed, it is known that rectal leiomyomas rarely recur. In a case series with a focus on clinical outcomes of endoscopic removal by Choi et al., 22 patients were diagnosed with polypoid leiomyomas; 19 of those patients underwent at least one follow-up, and no patients were identified to have local remnant or recurrent lesions [5]. In the absence of clear guidelines for follow-up of specific GI leiomyomas, patients should continue to receive routine colonoscopy per current screening guidelines for colon and rectal polyps.

Rectal leiomyomas have been reported to have a significant male predominance [5,9]. With a focus on rectal leiomyomas occurring in females, this case raises the question of whether there is any relationship between having uterine leiomyomas and concomitant GI or, more specifically, rectal leiomyomas. This co-occurrence has been rarely reported in current literature. The occurrence was reported once previously by Chan et al. in 2016, highlighting a case of a 47-year-old female who was found to have a rectal leiomyoma that co-occurred with multiple uterine myomas [9]. This relationship highlights a potential area for further investigation. Although GI leiomyomas are classically benign, large leiomyomas in the colon or rectum may present with complications similar to those seen with other large GI masses or even leiomyomas in the uterus with abdominal pain, bleeding, or obstruction.

## Conclusions

Rectal leiomyoma is a rare finding, typically discovered incidentally during the histologic examination of colonoscopy specimens. Accurate diagnosis relies on complete histopathologic analysis following resection. While this pathology is uncommon as a single entity, the co-occurrence of rectal leiomyoma in a female patient with uterine leiomyomas has been documented in the literature only once previously. This unusual presentation suggests a potential link between uterine and extrauterine leiomyomas.

Future research should investigate the significance of co-occurring rectal and uterine leiomyomas in females to better understand their possible connections and implications for clinical practice. Clinically, the detection of a leiomyoma during routine colonoscopy should prompt consideration of its implications for surveillance. In the absence of specific guidelines for follow-up after GI leiomyoma resection, the patient in this case will undergo a repeat colonoscopy in five years, consistent with the standard follow-up protocol for most other low-risk polyps.
